# Foot and ankle injuries during the Athens 2004 Olympic Games

**DOI:** 10.1186/1757-1146-2-9

**Published:** 2009-04-12

**Authors:** Thanos Badekas, Stamatios A Papadakis, Nikolaos Vergados, Spyros P Galanakos, Angeliki Siapkara, Mike Forgrave, Nick Romansky, Steven Mirones, Hans-Jeorg Trnka, Marino Delmi

**Affiliations:** 1 Olympic Village Polyclinic, Foot and Ankle Department, Health Services Athens 2004 Olympic Games, Athens, Greece

## Abstract

**Background:**

Major, rare and complex incidents can occur at any mass-gathering sporting event and team medical staff should be appropriately prepared for these. One such event, the Athens Olympic Games in 2004, presented a significant sporting and medical challenge. This study concerns an epidemiological analysis of foot and ankle injuries during the Games.

**Methods:**

An observational, epidemiological survey was used to analyse injuries in all sport tournaments (men's and women's) over the period of the Games.

**Results:**

A total of 624 injuries (525 soft tissue injuries and 99 bony injuries) were reported. The most frequent diagnoses were contusions, sprains, fractures, dislocations and lacerations. Significantly more injuries in male (58%) versus female athletes (42%) were recorded. The incidence, diagnosis and cause of injuries differed substantially between the team sports.

**Conclusion:**

Our experience from the Athens Olympic Games will inform the development of public health surveillance systems for future Olympic Games, as well as other similar mass events.

## Background

The Olympic Games represent the ultimate challenge for competitors. However, they are associated with a certain risk of injury for the participating players or athletes. Appropriate planning and staffing for medical services at large-scale athletic events is essential to provide for a safe and successful contest. Increased public health surveillance was first described for the 1984 summer Olympic Games in Los Angeles [1].

The XXVIII Olympic Games competition period held in Athens commenced on August 11, the Games declared open on August 13, and the period of competition that hosted 28 sports (swimming, diving, synchronised swimming and water polo are classified by the IOC as disciplines within the sport of aquatics, and wheelchair racing was a demonstration sport) concluded with the Closing Ceremony on August 29 2004. Eleven thousand and ninety-nine athletes competed, some 600 more than expected, accompanied by 5,501 team officials from 202 countries.

There were 301 medal events in 28 different sports. The Athens Organising Committee (AOC) was responsible for planning and delivery of both the Olympic and Paralympic Games. The AOC Medical Commission principles of protecting the health of athletes, respect of both medical and sport ethics, and equality for all competing athletes were a high priority for the establishment of the medical facilities for Athens 2004.

The Foot and Ankle Department was responsible for all lower limb, foot and ankle injuries encompassing bone, muscle, tendon, other soft tissue structures and skin conditions. An array of acute and chronic musculoskeletal conditions were investigated, diagnosed and treated, encompassing bone stress, tendonopathies, fasciitis and muscle dysfunction, biomechanical overload, the investigation of overload injuries and rectification of footwear issues.

The Foot and Ankle Department was not only present at the Polyclinic but also attended competition and training venues involving track and field, marathon, race walks, volleyball and basketball. In addition, access to other venues for sports that required lower limb injury surveillance and treatment, such as tennis, enabled the medical service to provide a comprehensive and cohesive multidisciplinary sports medicine approach. At these venues the Foot and Ankle Department worked with other members of the medical team to provide pre-event, intra-event and immediate post-event care for all athletes. When necessary more extensive investigation and treatment was referred to the Polyclinic.

The purpose of this study was to report foot and ankle injuries at the 2004 Athens Olympic Games and to assist in the planning of similar events in the future.

## Methods

### Medical Organisation

The medical organisational structure was directed by 5 managers: a Medical Manager for the Competition Venues, a Medical Manager for the Non-Competition Venues, a local Medical Director for the Polyclinic, a Complex Venue Medical Manager, and a Liaison Manager (for the Hospitals, Emergency Medical System (EMS), Public Health, Supplies, and Staffing). The health services personnel consisted of 5,210 individuals. Of those, 1120 were Health Care Providers (including 360 Medical Doctors (MD), 480 Nurses, 180 Massage Therapists, 40 Dentists, 30 Opticians and 30 Podiatrists), 760 were employees of hospitals (first aid, EMS), 120 were responsible for public health/hygiene, and the 200 others were administrative personnel. The role of 3,010 volunteers was also noteworthy.

The Polyclinic in the Olympic Village was a very important structural part of the medical organisation. The Polyclinic had an emergency ward (which was supported by ambulance services), outpatient services (Internal Medicine, Orthopaedics, Foot and Ankle, Ear-Nose-Throat, Dermatology, Gynaecology, Cardiology and Psychiatry), short-term observation room, Dentistry, Physical Therapy-Rehabilitation, Imaging Department, Laboratory and Pharmacy. Although the polyclinic was available to all athletes, teams from some countries had their own physicians and other medical personnel. These medical teams were provided separate space within their residential areas. In addition, there were Health Care Interpreters employed by the Polyclinic, medical staff for doping control and IOC Medical Commission staff for medication notification.

### Structure and functioning of the Foot and Ankle Department

The delivery of foot and ankle care for Athens 2004 commenced in August 2001. Over a period of three years the framework for foot and ankle surgeons' roles was established inclusive of; overseeing infrastructure building requirements, planning of athlete care, sourcing inventory such as medical equipment and consumables, development of administration procedures, selection of a team, rostering of work shifts, billeting of interstate orthopaedic surgeons, liaison with other medical disciplines to coordinate the most effective approach to athlete care, and liaison with medical teams from National Olympic Committees (NOCs) and National Paralympic Committees (NPCs).

The Foot and Ankle Department operated from the 31^st ^of July until the 30^th ^of August 2004 and it employed 36 staff; 5 of which were Orthopedic Surgeons specialised in Foot and Ankle Surgery, 15 were Podiatrists and 15 Certified Pedorthists (CPeds). The department included a reception area, two examination rooms, one pedobarograph machine and a laboratory for the construction of insoles and orthoses. It was open from 8 am to 10 pm and the operation was divided into two shifts. In each shift there was at least one MD with two Doctors of Podiatric Medicine and two to three CPeds. Interview rooms were also available for consultation with practitioner, athlete, radiologist and coach or medical officer of the patient when images were interpreted. A centralised sterilisation area was located within the Polyclinic that served all departments.

### Data collection

An observational, epidemiological survey was used to analyse injuries in all sport tournaments (men's and women's) over the period of the Games. To achieve this we retrospectively analysed the medical records of 624 patients that were consulted by the Foot and Ankle Department. For all types of injuries, the following information were documented: sex, age, injured body part and type of injury, circumstances (noncontact, contact, foul play), and consequences of injury (referee's sanction, treatment, time-loss in sport). Because follow-up was not possible, the physicians were asked to state an estimate of the duration of the player's likely absence from training and/or matches as a result of the injury. All team sports, as well as the athletes which were included in this report, followed the same methodology. Information was collected from the logbooks and medical encounter forms. Data collection started when the Olympic Village was open and lasted until the end of the Olympic Games.

## Results

**Figure 1:**
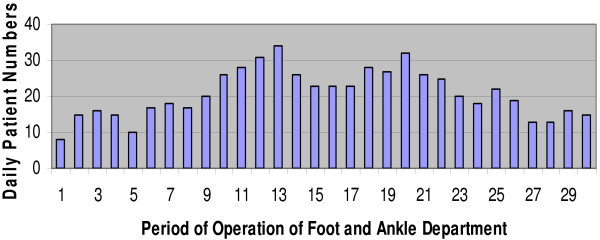
**Frequency of consultations over the study period**.

During the Olympic period August 1^st ^to September 1^st^, 624 patients presented to the Foot and Ankle Department for treatment. The mean age of athletes was 24 years (range 21 to 32), whereas the mean age of the media, Olympic family and officials was older at 42 years (range 28 to 57). Among the patients there were more males, 358 (58%) than females, 266 (42%). A detailed breakdown of the injuries sustained is included in Table 1. Athlete medical encounters represented 64% of the patients, personnel 18%, coach 7%, IOC family 6%, and 5% were classified as other. 84.1% of consultations were cases relating to musculoskeletal injury. The rest (15.9%) related to primary care issues, which involved the treatment of skin and nail conditions, and diabetic care. Figure 1 shows the frequency of consultations on each day of operation of the Foot and Ankle Department.

**Table 1:** Characteristics of foot and ankle injuries in sport tournaments during the Athens 2004 Olympic Games.

	**No. of injured athletes**		**No. of all injuries according to sport**							
	Total	Male/Female	Soccer M/F	Handball M/F	Basketball M/F	Apparatus work – Gymnastic M/F	Obstacle race M/F	Volleyball M/F	Weight lifting M/F	Horse – riding M/F

**Type of injury**	624									

***Soft tissue injuries***	525	365/160								

Achilles tendinitis	153	101/52	31/13	23/5	21/16	12/15	11/2	3/1	0/0	0/0

Ankle sprains	138	100/38	28/9	16/7	13/10	7/9	19/2	17/1	0/0	0/0

Peroneal tendinitis	64	48/16	12/3	8/2	7/4	4/3	9/2	5/1	3/1	0/0

Nail infections/injuries	52	39/13	11/2	6/1	7/3	3/5	5/1	5/1	0/0	2/0

Lesser toes sprains	45	31/14	9/2	8/3	10/6	2/3	0/0	2/0	0/0	0/0

Tibialis anterior tendinitis	28	19/9	4/1	3/0	2/1	3/2	2/3	1/1	4/1	0/0

Shin splints	13	8/5	0/0	1/0	0/0	0/0	3/3	0/0	0/0	4/2

Skin infections	11	7/4	2/0	1/0	2/1	0/0	1/2	1/1	0/0	0/0

Turf toe	8	5/3	2/1	0/0	1/1	0/1	0/0	2/0	0/0	0/0

Morton neuroma	6	2/4	0/0	1/1	0/1	0/1	0/1	0/0	1/0	0/0

Hind-foot sprains	5	3/2	1/1	0/0	0/0	0/1	2/0	0/0	0/0	0/0

Plantar plate rupture	2	2/0	0/0	0/0	1/0	0/0	0/0	1/0	0/0	0/0

***Bony injuries***	99	58/41								

Stress fractures	29	18/11	3/1	5/2	6/2	2/4	2/2	0/0	0/0	0/0

Hallux rigidus	21	18/3	3/0	2/0	5/1	0/1	6/1	2/0	0/0	0/0

Hallux valgus	18	2/16	0/3	0/5	1/6	1/2	0/0	0/0	0/0	0/0

Accessory bone injuries	9	7/2	2/0	0/1	3/0	1/0	1/1	0/0	0/0	0/0

Latelar malleolus fractures	7	4/3	1/0	1/2	1/0	0/0	1/1	0/0	0/0	0/0

Sesamoid fractures	3	2/1	1/0	0/0	0/0	1/0	0/0	0/1	0/0	0/0

5^th ^metatarsal tubercule fractures	3	2/1	1/0	0/1	1/0	0/0	0/0	0/0	0/0	0/0

Bimalleolar fractures	2	1/1	0/0	0/0	0/1	0/0	0/0	0/0	0/0	1/0

Pilon fractures	2	2/0	1/0	0/0	0/0	0/0	0/0	0/0	0/0	1/0

Freiberg's disease	2	0/2	0/0	0/0	0/0	0/1	0/0	0/0	0/1	0/0

Proximal phalanx hallux echondroma	1	1/0	0/0	0/0	1/0	0/0	0/0	0/0	0/0	0/0

**Total**			112/37	75/30	82/53	36/48	63/21	39/7	8/3	8/2

**Figure 2:**
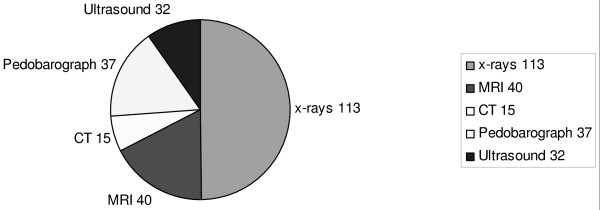
**Imaging technique that determined the diagnosis of the patients that presented to the Foot and Ankle Department for treatment**.

In 80% of consultations, acute management was carried out encompassing conditions such as onychocryptosis, paronychia and petechiae blisters. 218 acute injuries were treated (117 during event and 101 out of event), whereas 201 were old injuries and 25 were acute trauma on chronic preexisting problems. Essential to podiatric management was the availability of imaging facilities inclusive of plain films, magnetic resonance imaging, computerised tomography, diagnostic ultrasound and podobarograph (Figure 2).

Musculoskeletal injury (84.1%) included acute injury, overuse injury and injury due to biomechanical anomalies. The consultations encompassing acute injury were diverse and involved an array of conditions including: ankle sprains; foot, tibial and fibula fractures; tendon tears of the peroneals, tibialis posterior and Achilles; compartment syndromes; and fasciitis. Overuse injuries included; chronic exertion syndromes, anterior and medial tibial stress conditions, plantar fasciitis, patellofemoral dysfunction and general tendonopathies. Biomechanical rectification included the management of athletes that had completed competition or who were forced to retire from their event due to injury. Foot and leg biomechanics, inclusive of gait evaluation, were assessed to provide diagnostic information relating to leg length discrepancy, ankle equinus, tarsal coalition, other maladies and advice regarding running shoes.

In 525 (84.1%) patients there was only a soft tissue injury and in 99 (15.9%) patients there was bone involvement. Regarding specific diagnoses, tendinitis was the most common reason for a visit, followed by ankle sprains, nail infections/injuries, lesser toes sprains, and stress fractures. Sixty-nine (11%) required emergency transfer to the hospital. Diagnoses included fractures [proximal diaphyseal 5^th ^metatarsal (n = 2, 0.3%), 5^th ^metatarsal tubercule (n = 3, 0.4%), stress (n = 29, 4.6%)], skin infections (n = 11, 2%) and ankle sprains grade C (n = 24, 4.5%). Thirty-five cases (5.6%) suffered ankle fractures (lateleral malleolus, bimalleolar, Pilon) and ankle sprains (grade C), which underwent surgical treatment (Table 2).

**Table 2:** Age of athletes and injury characteristics

**Age (range)**	N (Males/Females)	%	
			

20–29 years	401 (286/115)	64.2	

30–40 years	223 (118/105)	35.8	

			

**Soft tissue injuries**	N		**Cases which required surgical treatment**

Tendinitis	245	39.2	

Ankle sprain	138	22.1	

Grade A	42	8	

Grade B	72	13.7	

Grade C	24	4.5	All

Nail infections/injuries	52	8.3	

Lesser toes sprains	45	7.2	

			

**Bony injuries**			

			

Stress fractures	29	4.6	

Lateral malleolus fractures	7	1.1	All

5^th ^metatarsal tubercule fractures	3	0.4	All

Bimalleolar fractures	2	0.3	All

Pilon fractures	2	0.3	

For all team sports, most injuries affected the lower extremity; 23.8% (n = 149) in soccer, 21.6% (n = 135) in basketball, 16.8% (n = 105) in handball, 13.5% (n = 84) in apparatus work – gymnastic and obstacle race, 7.3% (n = 46) in volleyball, 1.7% (n = 11) in weight lifting and 1.6% (n = 10) in horseriding. The type of injury was significantly different among team sports, with severe injuries, such as fracture and ligament injuries, more frequent in soccer, basketball, handball, obstacle race and volleyball compared to other sports (Table 3).

**Table 3:** The frequency and the types of injury by the team sports

	**Team Sports**				
	
	Soccer	Basketball	Handball	Obstacle race	Volleyball
**Type of injury**					

Ligament injuries (N/%)	105/20	92/17.5	75/14.2	52/9.9	34/6.4

Fractures (N/%)	11/11.1	13/13.1	12/12.1	9/9.1	1/1.1

The causes of injuries also varied substantially between the team sports. While 75% of gymnastic apparatus work and 57% of volleyball injuries occurred without contact, the majority of injuries in soccer (100%), handball (86%) and basketball (83%) occurred because of contact with another player or an object.

The number of foot orthoses dispensed during the Olympic period represented 30% of treatment that involved musculoskeletal injury. Ortho-mechanical treatment involving heel raises, heel cups, foot wedging, strapping and ankle bracing was also a significant component of the treatment program.

## Discussion

In 2004 the Olympic Games returned to Greece, the home of both the ancient Olympics and the first modern Olympics. For the first time ever a record 201 National Olympic Committees participated in the Olympic Games. The overall tally for events on the programme was 301 (one more than in Sydney 2000). Popularity in the Games reached new highs as 3.9 billion people had access to the television coverage compared to 3.6 billion for Sydney 2000. Planning for medical services at the Olympics began in Atlanta in 1991 [2,3].

In reviewing the literature on sports injuries, we found only a few studies in which exposure related incidences of injury in different types of sport were compared using the same methods [4-10]. Although all of these studies focus on injuries during a season, Cunningham and Cunningham [4] surveyed the incidence of injuries during the 1994 Australian University Games, a mass gathering event featuring 5,106 participants competing in 19 sports. The great advantages of conducting a comparative study during a sports tournament are that multiple sports with the players of a comparable skill level can be included and that the study period is defined by the event. Furthermore, in a top class international tournament, a high standard of environmental factors, such as the quality of the playing fields and equipment, is guaranteed.

However, a comparison with previous studies on injuries in team sports is difficult because of the methodological problems such as heterogeneous definitions of injury, study populations, methods of assessment, and calculations of incidence. Furthermore, detailed prospective studies on the incidence, type of injuries, and circumstances of injuries could not be found for all team sports included in the present study.

Most information is available about injuries of elite male [7,8] and female [11,12] soccer players, and these studies are in agreement with the present results. Handball injuries have also been investigated in several studies [13-15] but the reported incidences and characteristics of injury varied substantially. However, handball injury rates similar to our study have been reported from other tournaments [13,16] and in a retrospective study on self-reported injuries during a season [17]. In two prospective studies on basketball injuries [18,19], the rates of injury were lower than in the present study, probably because of the lower skill level of the players and/or standard of the tournaments. Nevertheless, the results in relation to location and diagnosis of injury were in agreement with our study. Two prospective studies on volleyball injuries are also in agreement with the present study [20,21].

Finally, in the Olympic Games, treatment modalities need to be oriented towards the most conservative and efficient options. Athletes who compete in such events usually prepare for a long time and in a very intense way. It is therefore appropriate that they be given every chance of being able to participate. Accordingly, improvement of any biomechanical abnormalities can make a substantial difference, thereby allowing them to compete when they may not have been able to otherwise.

## Conclusion

The 2004 Athens Olympic Games was a mass gathering with unique characteristics that created complex demands on medical service delivery. During this event, the risk of injuries in some team sports tournaments was higher than in others. Accordingly, prevention of injury and promotion of fair play are relevant issues for almost all team sports [22-24]. The experience of Athens Olympic Games will inform the development of public health surveillance system for future Olympic Games, as well as other similar mass events.

## Competing interests

The authors declare that they have no competing interests.

## Authors' contributions

TB, SAP, NV, SPG, AS, MF, NR, SM, H-JT, and MD, participated in the design of the study, data acquisition and analysis and writing of this manuscript. TB, NV and SPG, participated in the analysis and writing of this paper. TB, and SAP, participated in the analysis and also in revising the manuscript. All authors read and approved the final manuscript.
